# Differences in the Incidence of Sterile Inflammation After Trabectedin Infusion With Two Central Venous Port Systems: A Retrospective Study

**DOI:** 10.7759/cureus.57507

**Published:** 2024-04-03

**Authors:** Takatoshi Kubo, Koichiro Yasaka, Hiroshi Kobayashi

**Affiliations:** 1 Radiology, The University of Tokyo Hospital, Tokyo, JPN; 2 Orthopedic Surgery, The University of Tokyo Hospital, Tokyo, JPN

**Keywords:** sarcoma soft tissue, sterile inflammation, central venous port, central venous access, trabectedin

## Abstract

Purpose

Sterile inflammation along the tunneled catheter is a characteristic complication associated with trabectedin infusion via a central venous port (CVP). To date, no studies have evaluated the differences in sterile inflammation incidence according to the CVP system used. This study evaluated the differences in sterile inflammation incidence between two different CVP systems.

Methods

This study was conducted at The University of Tokyo Hospital, Bunkyo-Ku, Tokyo, Japan. Patients with trabectedin infusion using CVP via the internal jugular vein between April 2016 and February 2024 were retrospectively evaluated. Sterile inflammation was characterized as skin erythema, swelling, pain, or induration along the tunneled catheter after infusion of trabectedin from the CVP and negative for various infection tests. The incidence of sterile inflammation was compared using two different CVP systems: Anthron® polyurethane catheter with Celsite port (P-U Celsite; Toray Medical, Tokyo, Japan) and DewX Eterna (Terumo, Tokyo, Japan).

Results

Of the 21 patients, 12 and nine patients used P-U Celsite and DewX Eterna for trabectedin infusion, respectively. Sterile inflammation occurred in five patients; of these, four underwent CVP removal because of worsened pain, making trabectedin infusion difficult. Sterile inflammation occurred in 0 (0/12) and 56% (5/9) of patients using P-U Celsite and DewX Eterna, respectively, with a significantly lower incidence in patients using P-U Celsite (P = 0.006).

Conclusion

Sterile inflammation incidence was significantly lower in patients using P-U Celsite compared to those using DewX Eterna.

## Introduction

Trabectedin is an antineoplastic alkaloid with a multimodal mechanism of action that is used to treat advanced soft tissue sarcomas [1-6]. Trabectedin is administered as a 24-h continuous infusion, every three weeks [7-9]. As trabectedin is a vesicant drug, extravasation from the vessels damages the surrounding tissue [10]. Trabectedin leakage causes blistering, severe pain, and tissue necrosis, and debridement and skin reconstruction are necessitated in severe instances [10,11]. To reduce the risk of extravasation, trabectedin is administered via a central venous route, mainly central venous port (CVP) [12].

A multicenter retrospective study from the Netherlands reported typical sterile inflammation along the tunneled catheter pathway by trabectedin infusion from the CVP, mainly using Port-a-Cath® (Smiths Medical, Dublin, OH) [12]. The inflammation occurred predominantly along the catheter trajectory without evidence of infection and was believed to be caused by a small quantity of trabectedin permeating from the catheter porosity. Because the access route was not described in the previous report [12], the incidence of sterile inflammation with CVP via the internal jugular vein has not been elucidated. Additionally, no studies have compared differences in sterile inflammation incidence between the different CVP systems used.

This study aimed to evaluate differences in the incidence of sterile inflammation along the catheter in trabectedin infusion from the CVP via the internal jugular vein, according to the CVP system used.

## Materials and methods

Study design

This retrospective, observational study was conducted at The University of Tokyo Hospital, Bunkyo-Ku, Tokyo, Japan, and approved by its Ethics Review Board (approval #2561-(26)). This study included consecutive CVP implantations for trabectedin infusion performed at our institution between April 2016 and February 2024 by retrospectively analyzing medical records. Patients with CVP inserted via veins other than the internal jugular vein were excluded.

Written informed consent was obtained from all patients before the procedure. Because of the retrospective nature of the study, the requirement for additional informed consent for publication was waived.

CVP placement

During hospitalization, all procedures with local anesthesia on X-ray fluoroscopy tables equipped with portable ultrasonography being performed by 10 experienced interventional radiologists. The two CVP systems used at our hospital during the study period were a 6-F Anthron® polyurethane catheter with Celsite port (P-U Celsite; Toray Medical, Tokyo, Japan) and a 6-F DewX Eterna (Terumo, Tokyo, Japan). Operator preference determined which CVP was used. In all patients, the catheter was tunneled through the subcutaneous fat tissue from the port main body in the anterior thoracic to the vascular entry site in the ipsilateral internal jugular vein.

**Figure 1:**
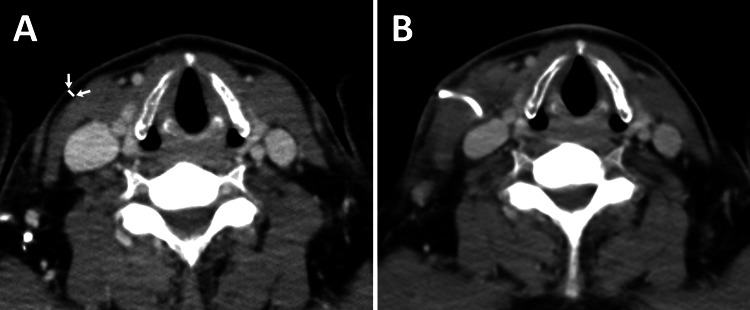
Cervical subcutaneous fat tissue thickness before central venous port implantation on CT A: Cervical subcutaneous fat tissue thickness of the subcutaneous catheter route was retrospectively evaluated on CT performed within one month pre-CVP implantation (white line between arrows). B: CT after CVP implantation.

Further, we retrospectively evaluated the cervical subcutaneous fat tissue thickness of the subcutaneous catheter route before CVP placement on CT performed within one month pre-CVP implantation (Figure 1).

Trabectedin infusion

Primarily, trabectedin was administered over 24 h from the CVP under hospitalization every three weeks. We confirmed no suspicious findings of massive trabectedin leakage during and immediately after infusion, such as swelling or acute skin reactions around the CVP site. Outpatient evaluations were performed between cycles as needed. Trabectedin was continued until disease progression, remission, or unacceptable toxicity occurred.

Definition and management of sterile inflammation

**Figure 2:**
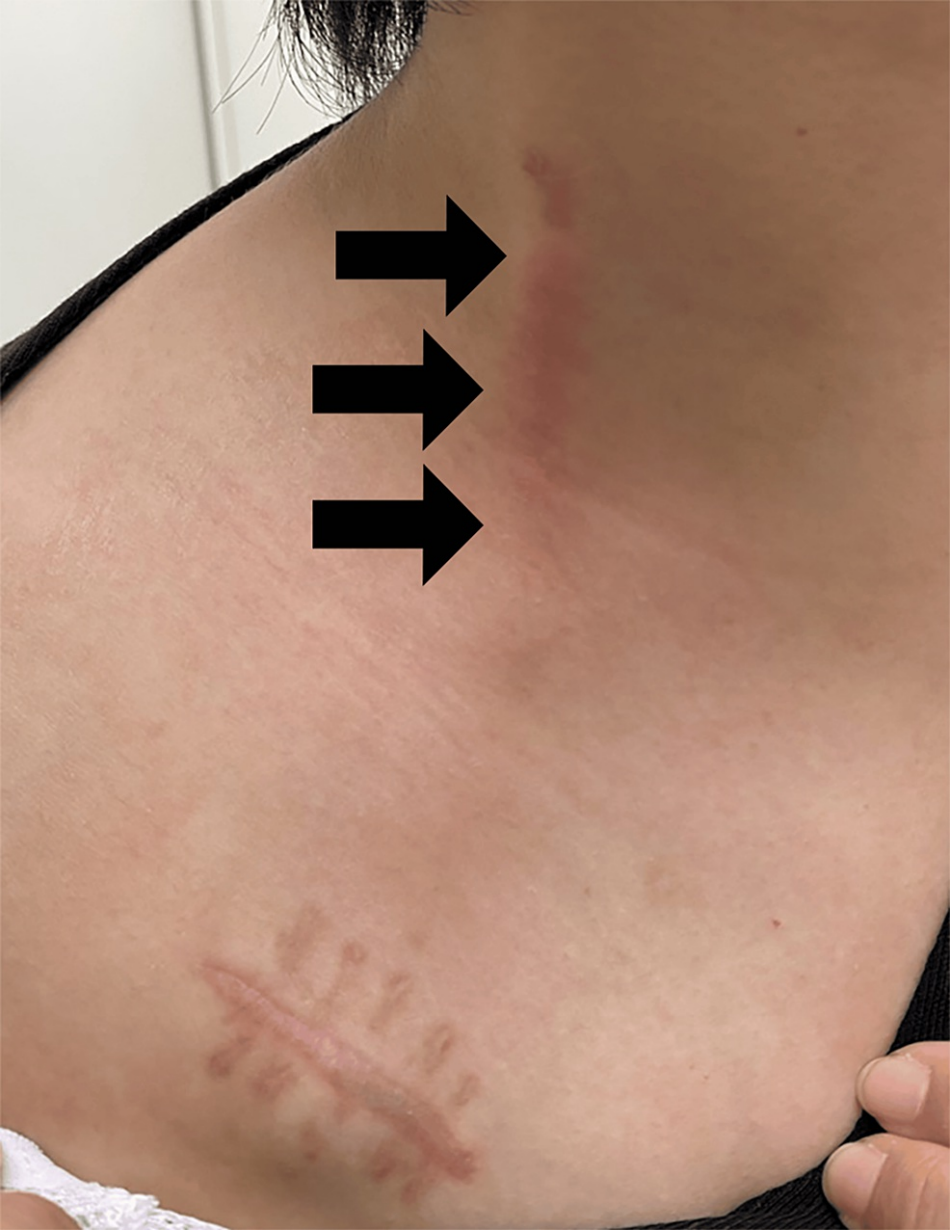
Sterile inflammation along the central venous port catheter trajectory A 34-year-old woman who had received three cycles of trabectedin infusion from the CVP (DewX Eterna) via the right internal jugular vein developed erythema and pain along the tunneled catheter (arrows) one week after the completion of the three cycles. As the culture was negative, the diagnosis of sterile inflammation was made, and steroid ointment was applied. However, the patient was unable to continue trabectedin infusion from the CVP because of worsening pain, and the CVP was eventually removed.

Sterile inflammation was characterized as skin erythema, swelling, pain, or induration along the subcutaneous tunneled catheter after trabectedin infusion from the CVP without detecting the etiologic microorganism responsible for the symptoms by bacterial cultures. A typical case of sterile inflammation is shown in Figure 2.

Statistics analysis

Patient characteristics and outcomes were summarized. Sterile inflammation incidences were compared using Fisher’s exact test by the CVP system. All P values were two-tailed, and P <0.05 was considered statistically significant. Statistical analyses were performed with R (version 4.1.2; R Development Core Team, Vienna, Austria).

## Results

Twenty-two patients underwent CVP placement for trabectedin infusion during the study period. One patient was excluded from the analysis because of CVP via the subclavian vein. Overall, 12 and nine patients used P-U Celsite and DewX Eterna, respectively. There were 10 males and 11 females, with their mean age being 54 ± 15 years (range, 30-73). The mean thickness of cervical subcutaneous fat tissue on CT pre-CVP placement was 2.6 ± 1.4 mm (range, 0.8-5.4). Patient details are shown in Table 1. No severe skin complications that required debridement or skin reconstruction were observed.

**Table 1:** Patient characteristics Except where indicated, data are means ± standard deviations, with ranges in parentheses *Data are numbers of patients, with percentages in parentheses; CVP, central venous port

Parameter	All Patients (n = 21)	P-U Celsite (n = 12)	DewX Eterna (n = 9)
Age, y	54 ± 15 (30–73)	59 ± 13 (34–73)	47 ± 15 (30–72)
No. of men*	10 (48)	6 (50)	4 (56)
Body mass index	22.9 ± 3.9 (18–30.7)	23.2 ± 4.2 (18–30.7)	22.3 ± 3.5 (18.2–29.2)
Cervical subcutaneous fat thickness, mm	2.6 ± 1.4 (0.8–5.4)	2.8 ± 1.5 (0.8–5.4)	2.2 ± 1.2 (1.2–4.9)
Underlying disease*			
Synovial sarcoma	1 (5)	1 (8)	0 (0)
Rhabdomyosarcoma	2 (10)	1 (8)	1 (11)
Liposarcoma	10 (48)	4 (33)	6 (67)
Leiomyosarcoma	4 (19)	2 (17)	2 (22)
Solitary fibrous tumor/hemangiopericytoma	3 (14)	3 (25)	0 (0)
Other sarcomas	1 (5)	1 (8)	0 (0)
No. of patients with right-side CVP implantation*	17 (81)	9 (75)	8 (89)
No. of total cycles of trabectedin infusion	3.2 ± 2.9 (1–14)	3.2 ± 3.7 (1–14)	3.3 ± 1.7 (1–6)

Sterile inflammation

Sterile inflammation occurred in five patients (24%) overall. Two occurred after two cycles of trabectedin infusion, and three occurred after three cycles. All patients had erythema, swelling, and induration, and four of them had pain. In patients with sterile inflammation, conservative treatment such as ointment application was administered, and trabectedin infusion from the CVP was continued. These symptoms were relieved in between cycles; however, a few days after the following infusion, they often flared up. Since sterile inflammation was not yet recognized at our hospital, antibiotics were used in the first case even though blood culture results were negative. Antibiotics were not used in the other cases. Four patients with pain eventually had difficulty continuing trabectedin infusion because of worsening pain and underwent CVP removal. The remaining patient completed a scheduled five cycles of trabectedin infusion, although he experienced sterile inflammation after two cycles.

Sterile inflammation occurred in 0% (0/12) of patients using P-U Celsite and 56% (5/9) using DewX Eterna, with a significantly lower incidence in patients using P-U Celsite (P = 0.006). The number of sterile inflammation occurrences per total cycle administered was zero per 38 cycles in patients using P-U Celsite and five per 30 cycles in patients using DewX Eterna.

## Discussion

In this retrospective study, five patients with sterile inflammation along the tunneled catheter were among the 21 patients who received trabectedin infusion from the CVP via the internal jugular vein. Sterile inflammation did not occur in patients using P-U Celsite but in 56% of patients using DewX Eterna, with a significantly lower incidence in patients using P-U Celsite.

Verboom et al. reported sterile inflammation along the tunneled catheter in 45 of the 107 patients who received trabectedin infusion from the CVP, mainly using a Port-a-Cath® [12]. Erythema, discomfort, and inflammation around the catheter site were noted in a subanalysis of a phase 3 randomized trial comparing the efficacy of trabectedin and dacarbazine in advanced leiomyosarcoma/liposarcoma [10]. Although it is unclear whether trabectedin was administered via a CVP or a central venous catheter, these catheter site complications could indicate similar sterile inflammation. Similar sterile inflammation was observed in this study; therefore, sterile inflammation along the tunneled catheter may be a characteristic complication of trabectedin infusion from the CVP, even though no published reports have been made regarding such. As trabectedin is a vesicant drug, broad severe skin complications such as skin necrosis and ulceration are frequently reported because of trabectedin leakage [10,11,13]. In contrast, sterile inflammation was localized around the tunneled catheter, improved between the cycles, and re-aggravated after trabectedin infusion [12]. These clinical profiles of sterile inflammation differ from those of common trabectedin leakage. Although it has not been proven, sterile inflammation is hypothesized to be caused by minimal trabectedin leakage from the porous catheter at the tunneled catheter site [12]. Sterile inflammation is not a severe cutaneous complication and with no infection. Therefore, immediate removal of the CVP is not necessary when it occurs. However, four of five patients in this study who developed sterile inflammation ultimately required CVP removal because of escalating pain rendering them unable to sustain trabectedin infusion. Therefore, sterile inflammation needs to be considered a complication that may necessitate CVP removal.

Verboom et al. reported no instances of sterile inflammation detected when tunneled catheters were placed deeply [12]. In their study, it is unclear whether the increasing surrounding tissue pressure caused by deep insertion prevented sterile inflammation or whether sterile inflammation occurred but remained undetectable because of the subcutaneous tissue thickness. Although this method appears useful, the cervical subcutaneous fat tissue thickness in this study was <6 mm in all patients, suggesting that it may not be possible to place the catheter deep under the skin in Asian populations owing to their thin subcutaneous fat tissue. Contrastingly, in this study, even with CVP implanted via the internal jugular vein and in thin subcutaneous tissue, there was no single case of sterile inflammation in patients using the P-U Celsite, and the incidence of sterile inflammation was significantly lower than in patients using the DewX Eterna. This suggests that using P-U Celsite may reduce the incidence of sterile inflammation. The Anthron® catheter used in the P-U Celsite system is heparinized hydrophilic polymers with large amounts of heparin ionically bonded to the polymer matrix and shows long-term blood compatibility [14,15]. This characteristic feature of the catheter may have influenced the incidence of sterile inflammation. Further studies are warranted to more precisely investigate the mechanism.

This study has some limitations. First, it was a small, retrospective study with selection bias and poor generalizability. Second, only two types of CVP, P-U Celsite and DewX Eterna, were used at our institution during the study period, and the incidence of sterile inflammation with other types of CVP remains unknown.

## Conclusions

Characteristic sterile inflammation along the tunneled catheter occurred in 56% of patients with trabectedin infusion from the CVP using DewX Eterna via the internal jugular vein. The incidence of sterile inflammation was significantly lower in patients who used P-U Celsite compared with those who used DewX Eterna.

Our findings support the use of P-U Celsite in patients receiving trabectedin infusion from CVP via the internal jugular vein.
